# Gintonin-Enriched *Panax ginseng* Extract Induces Apoptosis in Human Melanoma Cells by Causing Cell Cycle Arrest and Activating Caspases

**DOI:** 10.3390/foods14030381

**Published:** 2025-01-24

**Authors:** Su-Hyun Lee, Gyun-Seok Park, Rami Lee, Seongwoo Hong, Sumin Han, Yoon-Mi Lee, Seung-Yeol Nah, Sung-Gu Han, Jae-Wook Oh

**Affiliations:** 1 Department of Stem Cell and Regenerative Biotechnology, Konkuk University, Seoul 05029, Republic of Korea; skiara1122@gmail.com (S.-H.L.); hsw2332@naver.com (S.H.); hsm534@naver.com (S.H.); dldbsal1414@naver.com (Y.-M.L.); 2 Department of Bio-Resources and Food Science, Konkuk University, 120 Neungdong-ro, Gwangjn-gu, Seoul 05029, Republic of Korea; bhs2945@hanmail.net; 3 Ginsentology Research Laboratory and Department of Physiology, College of Veterinary Medicine, Konkuk University, Seoul 05029, Republic of Korea; rmlee12@konkuk.ac.kr (R.L.); synah@konkuk.ac.kr (S.-Y.N.); 4 Department of Food Science and Biotechnology of Animal Resources, Konkuk University, Seoul 05029, Republic of Korea

**Keywords:** gintonin-enriched fraction, human melanoma, cell cycle, apoptosis, xenograft mouse model

## Abstract

Gintonin, a non-saponin glycolipoprotein from *Panax ginseng*, acts as a lysophosphatidic acid ligand. However, its anticancer effects, especially in melanoma, remain unclear. This study investigated the anti-proliferative effects and intracellular signaling mechanisms of a gintonin-enriched fraction (GEF) from *Panax ginseng* in human melanoma cell lines. In vitro, GEF treatment significantly inhibited cell proliferation, reduced clonogenic potential, and delayed wound healing in melanoma cells. Flow cytometry and terminal deoxynucleotidyl transferase dUTP nick-end labeling (TUNEL) staining showed that GEF induced apoptosis, as evidenced by increased apoptotic cell populations and nuclear changes. GEF also caused cell cycle arrest in the G_1_ phase for A375 cells and the G_2_/M phase for A2058 cells. It triggered apoptotic signaling via activation of caspase-3, -9, poly (ADP-ribose) polymerase cleavage, and downregulation of B cell lymphoma-2 (Bcl-2). GEF treatment also raised intracellular reactive oxygen species (ROS) levels and mitochondrial stress, which were mitigated by N-acetyl cysteine (NAC), an ROS inhibitor. In vivo, GEF suppressed tumor growth in A375- and A2058-xenografted mice without toxicity. These findings suggest that GEF from *Panax ginseng* has potential antitumor effects in melanoma by inducing apoptosis and cell cycle arrest, presenting a promising therapeutic avenue.

## 1. Introduction

Melanoma is an aggressive and highly lethal form of skin cancer, accounting for the majority of deaths related to skin cancer globally [1,2]. Despite representing a smaller percentage of total skin cancer cases, its rapid progression, strong metastatic capability, and resistance to conventional treatments make it a significant clinical challenge [3]. The worldwide incidence of melanoma continues to rise, driven by environmental, genetic, and lifestyle factors [4]. This emphasizes the critical need to understand its underlying mechanisms and to develop more effective therapeutic solutions.

*Panax ginseng* has been extensively studied for its medicinal properties, including its anti-inflammatory, neuroprotective, immune-modulating and antitumor activity [5,6,7]. These benefits are largely due to its bioactive components, such as ginsenosides and gintonin [8,9]. Among these, the gintonin-enriched fraction (GEF), a specialized extract from *Panax ginseng*, contains lysophosphatidic acid (LPA) as a key active ingredient [10,11]. GEF exerts biological effects by interacting with LPA receptors, contributing to neuroprotection [12,13,14], modulation of immune responses [15], and anticancer activity [16]. This study has shown that GEF can inhibit melanoma cell metastasis and promote apoptosis by modulating intracellular calcium levels and regulating cell cycle and apoptosis pathways. It has also demonstrated efficacy in enhancing apoptosis in renal carcinoma cells [16] and addressing conditions such as sarcopenic obesity [17,18]. Due to its natural origin and low toxicity, GEF shows potential as a complementary agent in cancer treatment, particularly when combined with existing therapies to enhance their effectiveness. Further studies are needed to clarify its clinical applications.

The dysregulation of cell cycle and apoptosis mechanisms plays a key role in cancer progression, making them critical targets for therapeutic development [19]. Cancer cells often bypass normal cell cycle controls, such as G_1_/S (Gap1/Synthesis) or G_2_/M checkpoints, through alterations in cyclins, cyclin-dependent kinases (CDKs), and related regulators, enabling unchecked growth [20]. Simultaneously, they evade programmed cell death by suppressing caspase activity or overexpressing anti-apoptotic proteins [21]. Therapeutic approaches that inhibit cell cycle progression, such as CDK inhibitors, or restore apoptotic signaling via caspase activation have shown promising outcomes in reducing tumor growth and overcoming treatment resistance [22]. Combining strategies that target both pathways offers a promising avenue for improving cancer treatment outcomes [23].

This study explores the mechanisms through which GEF exerts its anticancer effects on melanoma, specifically its ability to regulate the cell cycle and induce apoptosis. Results indicate that GEF can arrest the cell cycle at G_1_ or G_2_/M phases and trigger apoptosis through mitochondrial dysfunction and caspase activation. These findings support the potential of GEF as a novel therapeutic candidate for melanoma treatment.

## 2. Materials and Methods

### 2.1. Gintonin-Enriched Fraction (GEF) Preparation

The GEF was obtained through extraction of *Panax ginseng,* followed by lyophilization, according to the methods outlined in a previous study [16,24]. In brief, locally sourced 4-year-old white ginseng (1 kg) was ground to pieces larger than 3 mm and refluxed with 70% ethanol for 8 h at 80 °C, repeated four times. This process yielded 333–350 g of concentrated ethanol extract. The extract was then dissolved in cold distilled water in a 1:10 ratio and stored at 4 °C for 24 h to allow precipitation. Following centrifugation at 3000 rpm for 20 min, the supernatant and precipitate were separated. The precipitate was freeze-dried and designated as GEF [16,24]. The resulting lyophilized GEF powder sample was stored at −80 °C and reconstituted in dimethyl sulfoxide (DMSO) prior to biological experimentation.

### 2.2. LC-MS/MS-Based Quantification of Lysophospholipids (LPLs) and Phospholipids (PLs) in GEF

Lysophospholipids (LPLs) and phospholipids (PLs) were quantified using the method of Hong and Cho et al. [14,25]. For a detailed lipid profile analysis, a 100 mg GEF sample in a 50 mL centrifuge tube was dissolved with 10 mL methanol and vortex-mixed. One milliliter of the layer was transferred to a new centrifuge tube and further diluted with methanol. The mixture was vortexed and then filtered through a 0.2 μm syringe filter (Millex-LG; Merck Millipore Corporation, Merck KGaA, Darmstadt, Germany). A volume of 2 μL was injected for LC-MS/MS analysis [25]. Stock solutions of LPLs and PLs were prepared in methanol and stored at 4 °C. Quantification was performed on an Agilent 1100 HPLC and API 2000 LC-MS/MS system in MRM mode using XBridge C18 and Hydrophilic Interaction columns with acetonitrile-based mobile phases. Electrospray ionization was conducted in positive mode for LPC/PC and negative mode for LPA, LPE, LPI, PA, and PI. Optimized conditions included specific voltages (−3.5 to −4.5 kV for negative, 5.5 kV for positive), a capillary temperature of 400–450 °C, and appropriate gas flows. Analyte quantification used MRM and an internal standard method based on peak area ratios [14,25].

### 2.3. Antibodies

The following antibodies (Abs) were utilized for detection: caspase-3 and caspase-9 (#9662S and #9508S; Cell Signaling Technology, Danvers, MA, USA), B cell lymphoma-2 (Bcl-2) (#H2813; Santa Cruz Biotechnology, Dallas, TX, USA), poly (ADP-ribose) polymerase (PARP) (#9542S; Cell Signaling), and β-actin (#D0618; Santa Cruz). Secondary antibodies included anti-mouse IgG or anti-rabbit IgG conjugated with horseradish peroxidase (HRP) (#1721011; Bio-Rad, Hercules, CA, USA and #2313567; Jackson ImmunoResearch, West Grove, PA, USA, respectively).

### 2.4. Cell Culture

The A375 and A2058 human melanoma cell lines were obtained from ATCC (Manassas, VA, USA) and cultured in Dulbecco’s Modified Eagle medium (DMEM) plus 10% fetal bovine serum (FBS), 10,000 units/mL penicillin, and 10,000 μg/mL streptomycin (Welgene, Daegu, Republic of Korea). The cultures were maintained in a humidified incubator at 37 °C with 5% CO_2_ and sub-cultured every three days for fewer than 15 passages before treatment [26].

### 2.5. Cell Viability Assay

Melanoma cell viability was evaluated by the 4-[3-(4-Iodophenyl)-2-(4-nitrophenyl)-2H-5-tetrazolio]-1,3-benzene disulfonate (WST-1) assay using an EZ-Cytox kit (DoGen, Seoul, Republic of Korea) [27]. The cells were seeded at a density of 3 × 10^3^ cells/well in 96-well microplates. Following overnight incubation, the cells were subjected to varying concentrations of GEF (0, 12.5, 25, 50, 100 and 200 μg/mL) or DMSO (control) in DMEM supplemented with 1% FBS for 24, 48, and 72 h. Then, each well received 10 μL of EZ-Cytox solution, followed by incubation at 37 °C for 1 h, and the optical density was measured at 450 nm with a reference wavelength of 600 nm using a spectrophotometer (SpectraMax M2e; Molecular Devices, San Jose, CA, USA) [26].

### 2.6. Colony Formation Assay

The cells were plated at a density of 1.5 × 10^5^ cells/well in 6-well plates. The following day, the cells were treated with specified GEF concentrations dissolved in DMEM supplemented with 1% FBS for 24 h. Following treatment, the cells were trypsinized, and 4 × 10^2^ cells from each group were seeded in 24-well plates for colony formation over 10 days. The colonies were fixed with methanol, stained with 0.5% crystal violet (JUNSEI, Tokyo, Japan), and imaged [28]. Quantitative analysis was conducted by extracting the dye with 10% acetic acid and measuring the absorbance at 590 nm using a spectrophotometer (SpectraMax M2e) [29].

### 2.7. Wound-Healing Assay

The cells were plated at a density of 2.5 × 10^5^ cells/well in 6-well plates. After an overnight incubation period, scratches were made on the cell monolayer using sterile 200 μL pipette tips, followed by washing with fresh DMEM supplemented with 1% FBS to create wounds. Subsequently, the cells were treated with specific concentrations of GEF in DMEM supplemented with 1% FBS for 24 or 48 h [28]. The extent of scratch closure in each group was captured and imaged using a light microscope (OLYMPUS CKX41, Shinjuku-ku, Tokyo, Japan), and quantified using ImageJ software (ImageJ1.53k_Java 1.8.0_172), as previously described [30].

### 2.8. Cell Cycle Assay

The cells were plated at a density of 1.5 × 10^5^ cells/well in 6-well plates. Following overnight incubation, the cells were treated with the specified amount of GEF in DMEM supplemented with 1% FBS for 24 h. Subsequently, the cells were collected, fixed in 70% ethanol at −20 °C for 16 h, washed with ice-cold Dulbecco’s Phosphate-Buffered Saline (DPBS), and then incubated with 100 μg/mL RNase A (Thermo Fisher Scientific, Waltham, MA, USA) and 50 μg/mL propidium iodide (PI) (BD Biosciences, San Diego, CA, USA). Cell cycle distribution was analyzed using a NovoCyte 1000 flow cytometer, and data were processed using NovoExpress 1.2.5 software (ACEA Biosciences, San Diego, CA, USA) [31].

### 2.9. Terminal Deoxynucleotidyl Transferase-Mediated dUTP Nick-End Labeling (TUNEL) Staining

The cells were seeded at a density of 8 × 10^3^ cells/well in 96-well plates. Following overnight incubation, the cells were exposed to specific concentrations of GEF in DMEM supplemented with 1% FBS for 24 h. Subsequently, the cells were rinsed with DPBS (Welgene) and fixed with 4% paraformaldehyde. Following fixation, the cells were washed with DPBS and permeabilized with PBST (0.2% Triton-X 100) [32]. TUNEL (Promega, Madison, WI, USA) and 4,6-diamidino-2-phenylindole, dihydrochloride (DAPI) (Sigma-Aldrich, St. Louis, MO, USA) staining were then applied to the cells. Cell morphology images were acquired using an ECLIPSE Ts2R inverted microscope (Nikon, Melville, NY, USA) and captured using NIS-Elements BR 5.01 software (Nikon) [16].

### 2.10. Apoptosis Assay

The cells were plated at a density of 1.5 × 10^5^ cells/well in 6-well plates. Following overnight incubation at 37 °C, the cells were exposed to varying concentrations of GEF in DMEM supplemented with 1% FBS for 24 or 48 h. Subsequently, the cells were trypsinized, collected, and subjected to apoptosis analysis using an FITC-Annexin V Apoptosis Detection Kit (BD Biosciences). Flow cytometric analysis was performed using a NovoCyte 1000 flow cytometer, and the data were analyzed using NovoExpress 1.2.5 software (ACEA Biosciences) [33].

### 2.11. Reactive Oxygen Species (ROS) and Mitochondrial Membrane Potential (MMP) Assay

Initially, the cells were plated at a density of 1.5 × 10^5^ cells/well in 6-well plates. Following overnight incubation, the cells were exposed to different concentrations of GEF in DMEM supplemented with 1% FBS for 24 and 48 h. GEF-induced ROS production in A375 and A2058 cells was assessed using the cell-permeable fluorogenic probe, 2′,7′-dihydro-fluorescein diacetate (DCFH_2_-DA), followed by flow cytometry.

The cells were subsequently plated at a density of 1.5 × 10^5^ cells/well in 6-well plates. Following overnight incubation, the cells were pre-treated with 4 mM N-acetyl cysteine (NAC; Sigma Aldrich) for 2 h prior to exposure to GEF at concentrations of 0, 75, and 150 μg/mL for 24 h for the ROS assay and 48 h for measuring MMP. For the ROS assay, the cells were supplemented with fresh medium containing 20 μM DCFH_2_-DA and incubated for 30 min at 37 °C, followed by a single wash with PBS and resuspension in 500 μL of PBS for flow cytometry [34].

For the MMP assay, the cells were treated with 2 μM JC-1 solution for 30 min at 37 °C and then examined using NovoCyte 1000 flow cytometer. The data were analyzed using NovoExpress 1.2.5 software (ACEA Biosciences) [35].

### 2.12. Western Blot Analysis

The cells were seeded in 60 mm dishes at a density of 3.5 × 10^5^ cells/well. Following overnight incubation, the cells were subjected to various GEF concentrations in DMEM containing 1% FBS for 48 h. Following treatment, the cells were lysed using RIPA buffer (Thermo Fisher Scientific). Equal amounts of protein (30 μg) from each sample were separated by sodium dodecyl–sulfate polyacrylamide gel electrophoresis (SDS-PAGE) and transferred onto polyvinylidene fluoride (PVDF) membranes (Bio-Rad) [29,36]. The membrane was incubated overnight at 4 °C with primary antibodies after being blocked with 5% skim milk against caspase-3, Bcl-2, PARP, and caspase-9, along with β-actin. Following incubation with an HRP-conjugated secondary antibody, the protein bands were visualized using chemiluminescence (Bio-Rad). The protein expressions were quantified by ImageJ software.

### 2.13. Xenografting of A375 and A2058 Cells

Xenografting of A375 and A2058 cells was performed using 24-week-old female athymic nude mice (Koateck, Pyeongtaek, Republic of Korea). The mice were divided into two groups, each with 10 mice per cell line. Subsequently, 1 × 10^6^ cells of A375 and A2058 suspended at a 1:1 ratio in Matrigel (Corning, Corning, NY, USA) were subcutaneously injected into the hind flank of each mouse [37]. All animal experiments were performed in accordance with the guidelines of the Institutional Animal Care and Use Committee (IACUC) of the Ethics Committee of Konkuk University (Seoul, Republic of Korea; IACUC No. KU23075). Once the tumors reached an approximate size of 100 mm^3^, the mice were divided into four groups, with three mice per group. These groups were orally administered GEF (150 mg/kg/day), whereas control mice were administered distilled water. Tumor size and body weight were monitored daily using digital calipers. The tumor volume was calculated using the formula V = (L × W^2)/2, where L is the tumor length and W is the tumor width. On day 21, tumor masses extracted from each group of mice were photographed [38].

### 2.14. Hematoxylin and Eosin (H and E) Staining and Terminal Deoxynucleotidyl Transferase dUTP Nick-End Labeling (TUNEL) Staining of Tumor Tissues

Tumor tissues were fixed in a 10% formalin solution (Thermo Fisher Scientific) and embedded in paraffin wax. After infiltration and after being embedded in paraffin, the tissues were sectioned and stained with H and E. Stained sections were examined under a light microscope (OLYMPUS CKX41) [39]. For TUNEL staining (Promega), to detect apoptotic cells within tumor tissues in accordance with the manufacturer’s protocol, tumor tissue sections were dewaxed and rehydrated. The sections were washed with PBS and permeabilized using Proteinase K (0.02 μg/μL). Subsequently, the sections were incubated with the TUNEL staining solution and treated with a mounting solution containing DAPI. The images were captured using an ECLIPSE Ts2R inverted microscope (Nikon) and analyzed using the NIS-Elements BR 5.01 software (Nikon) [16].

### 2.15. Statistical Analysis

The results are expressed as the mean ± standard deviation of three independent experiments, and statistical significance was assessed using a *t*-test or one-way ANOVA followed by Dunnett’s post-test (* *p* < 0.05, ** *p* < 0.01, *** *p* < 0.001 vs. control).

## 3. Results

### 3.1. Quantitation of Lysolipids (LPLs) and Phospholipids (PLs) in GEF Using LC-MS/MS

The lipid analysis of GEF contains ~11% free fatty acids (FFAs), with linoleic acid (C18:2) as the most abundant, followed by palmitic acid (C16:0) and oleic acid (C18:1), as previously reported [14]. GEF also includes 0.19% LPA C18:2 and smaller amounts of other lysolipids (LPC, LPE, LPI), totaling 0.52% LPLs. PLs account for 1.84%, mainly phosphatidic acids (PAs), including 1.17% PA 16:0–18:2, while PE, PG, and PS were not detected. Overall, GEF’s total lipid content is ~13.7%, with linoleic acid, LPA C18:2, and PA 16:0–18:2 as the key components (Supplementary Table S1).

### 3.2. GEF Suppresses Melanoma Cell Proliferation, Migration, and Colony Formation

**Figure 1:**
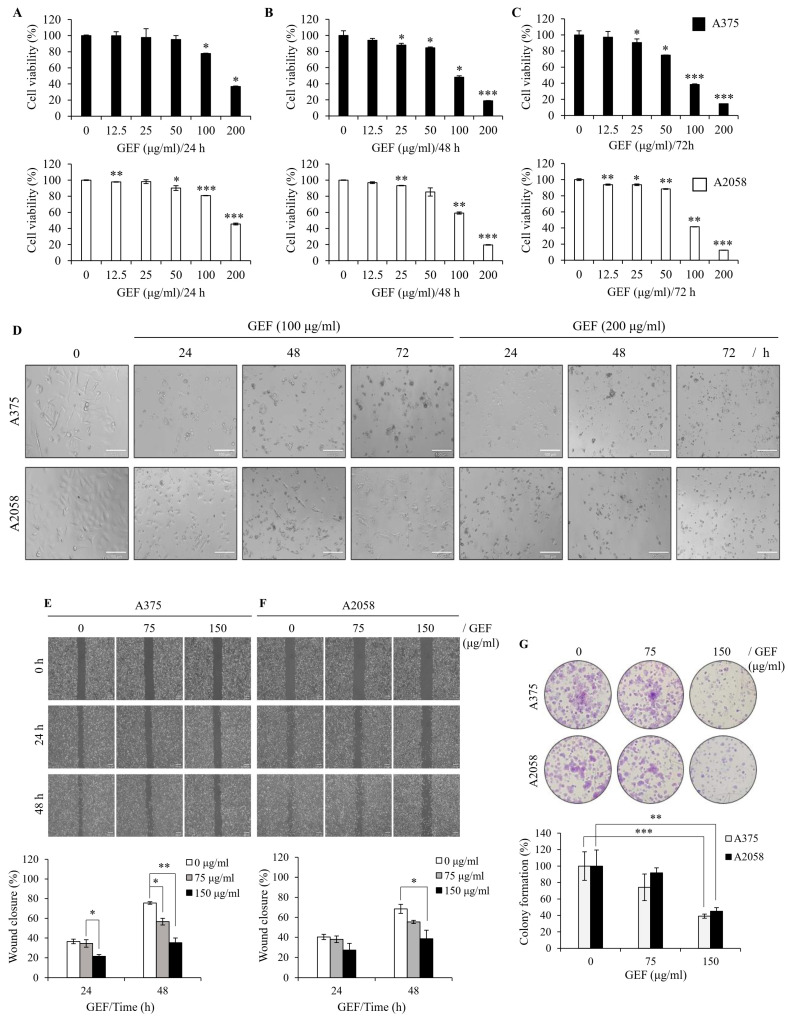
GEF suppresses cell proliferation, migration, and colony formation in melanoma cells. (**A**–**C**) A375 and A2058 cells were subjected to increasing concentrations of GEF (0–200 μg/mL) for 24–72 h. Cell viability was assessed using the WST-1 assay from three independent experiments. * *p* < 0.05, ** *p* < 0.01, and *** *p* < 0.001 compared to controls. (**D**) Phase-contrast microscopy revealed morphological alterations in cells treated with GEF (0, 100, and 200 μg/mL) for 24–72 h. Scale bar = 100 μm. (**E**,**F**) Cells were scratched and treated with GEF (0, 75, and 150 μg/mL) for 24–48 h. Relative migration inhibition was quantified compared to controls. (**G**) Cells were exposed to GEF at concentrations of 0, 75, and 150 μg/mL for 24 h, and colony formation was assessed after 10 days. Colony formation rates were compared to controls. Data represent the mean ± SD of three independent experiments. * *p* < 0.05, ** *p* < 0.005, and *** *p* < 0.001.

To examine the effect of GEF on melanoma cell survival, A375 and A2058 cells were treated with varying concentrations of GEF (0–200 μg/mL) for 24–72 h, and cell viability was measured using the WST-1 assay. Exposure to 0, 12.5, 25, 50, 100, and 200 μg/mL GEF induced a notable reduction in cell viability compared with that of the control group (Figure 1A–C), with survival rates of 14.5–90.5% for A375 cells and 12.4–93.6% for A2058 cells. Morphological changes, including increased cell death, were also evident in GEF-treated cells compared with those in the control group (Figure 1D). These findings indicate that GEF affects the viability of both melanoma cell lines.

To assess the effect of GEF on cell migration, a wound-healing assay was conducted on GEF-treated A375 (Figure 1E) and A2058 (Figure 1F) cells. The rate of wound closure was markedly diminished in GEF-treated cells compared with the control group for both cell lines. Specifically, exposure to 150 μg/mL GEF for 48 h resulted in a wound closure rate of 35.2% (A375 cells) and 38.6% (A2058 cells), compared with the control group with rates of 75.6% and 68.4%, respectively. These findings indicate significant inhibition of cell migration in both A375 and A2058 cells following GEF treatment.

To examine the effect of GEF on cell proliferation, a clonogenic assay was performed using both melanoma cell lines. Treatment with 75–150 μg/mL GEF significantly reduced the formation of new clones compared with the control (Figure 1G). The cloning efficiency (%) was notably decreased in A375 and A2058 cells (35.1% and 45.1%, respectively) following treatment with 150 μg/mL GEF. These findings indicate that GEF inhibits cell proliferation and migration in both human melanoma cell lines.

### 3.3. GEF Modulates Cell Cycle Arrest in Melanoma Cells

**Figure 2:**
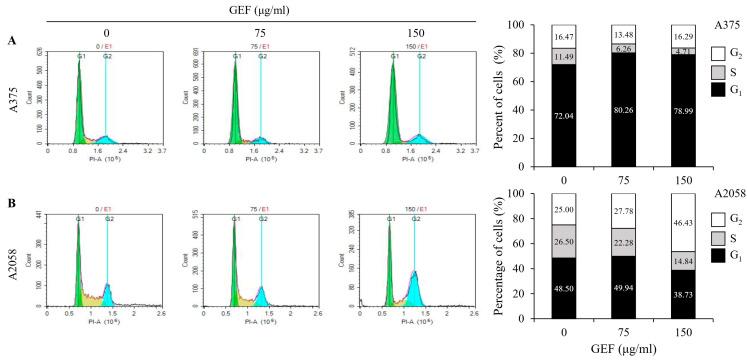
GEF induces G1 phase arrest in A375 cells and G2/M phase arrest in A2058 melanoma cells. The cells were treated with GEF (0, 75, and 150 μg/mL) for 24 h. The distribution of cells across different phases of the cell cycle was assessed after staining with PI and analyzed by flow cytometry. The histograms show the percentage of cells in each phase of for (**A**) A375 and (**B**) A2058. The data are representative of three independent experiments.

GEF treatment induced cell cycle arrest in A375 and A2058 cells (Figure 2A,B). Flow cytometric analysis revealed a dose-dependent increase in the proportion of cells in the G_1_ phase (80.26% and 78.99%, respectively) and a decrease in the S phase cells (6.25% and 4.71%, respectively) in A375 cells compared with those in the control. Conversely, in A2058 cells, the G_2_ phase cells significantly increased (46.43%), while the S phase cells decreased (14.84%) after treatment with GEF at 150 μg/mL. These findings suggest that GEF induces cell cycle arrest, with G_2_ phase arrest in A2058 cells and G_1_ phase arrest in A375 cells.

### 3.4. GEF Induces Apoptosis and Mitochondrial Stress in A375 and A2058 Cells

**Figure 3:**
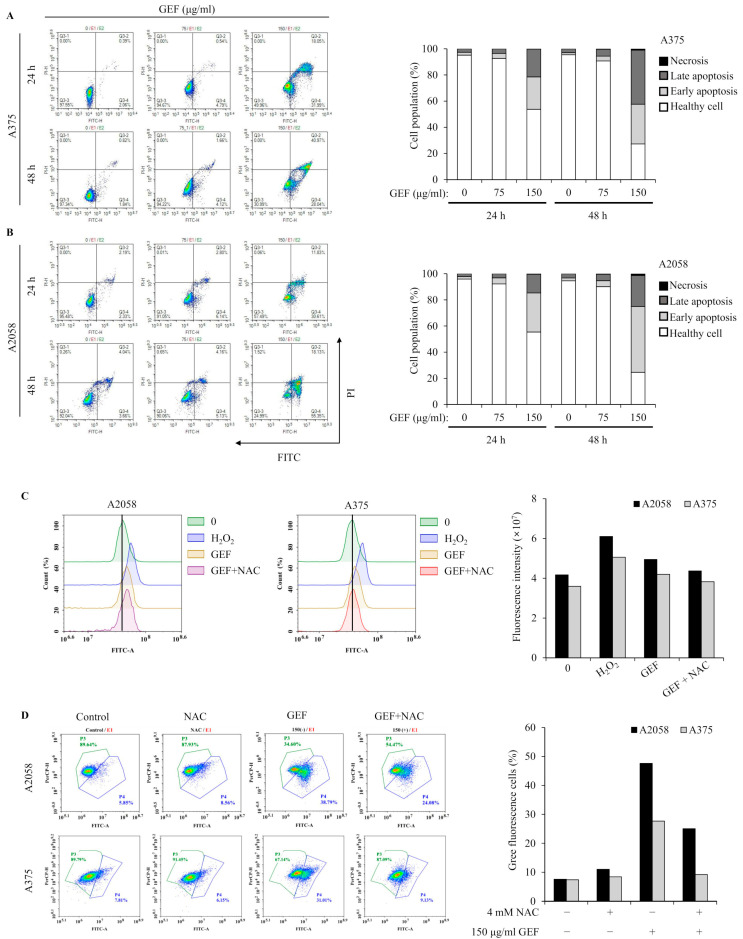
GEF prompts apoptosis in A375 and A2058 melanoma cells through ROS generation and MMP reduction. The cells were treated with GEF (0, 75, and 150 μg/mL) for 24 h. (**A**,**B**) Annexin V-FITC/PI co-staining was performed, and flow cytometry was used to assess the early/late apoptotic cell population. Cell numbers in each quadrant were counted and interpreted: (Q1) Necrosis, (Q2) late apoptosis, (Q3) healthy cells, and (Q4) early apoptosis. Histograms show quantitative cell percentage results for (**A**) A375 and (**B**) A2058. The data are representative of three independent experiments. (**C**) A375 and A2058 cells were pre-incubated with 4 mM NAC for 1 h prior to treatment with GEF (150 μg/mL) for 24 h. ROS production was measured using the DCFH_2_-DA method and flow cytometry. The histogram (right) shows quantitative fluorescence intensity. (**D**) After treatment with NAC, GEF, or both, changes in mitochondrial membrane potential (MMP) were detected using the JC-1 fluorescent probe. The histogram (right) shows the percentage of cells with green/red fluorescence. The data are representative of three independent experiments.

Apoptosis induction by GEF was investigated using annexin V/PI staining and flow cytometry. The results showed increased apoptotic cell populations in quadrants Q3-4 and Q3-2 for both A375 and A2058 cells following GEF treatment (150 μg/mL) for 24 h and 48 h (Figure 3A,B). Elevated levels of early and late apoptotic cells were observed in both cell lines. TUNEL staining revealed green fluorescent apoptotic cells in GEF-treated cells, indicating nuclear morphological changes and DNA fragmentation (Supplementary Figure S1).

Excessive ROS production in melanoma cells is associated with apoptosis induction [22]. GEF-treated cells showed increased DCF fluorescence over time, indicating elevated ROS levels, which were significantly reduced in the presence of NAC, an ROS inhibitor (Figure 3C,D). Mitochondrial function was also assessed, revealing GEF-induced mitochondrial stress, as evidenced by the changes in MMP. NAC significantly attenuated this effect, highlighting the involvement of GEF in ROS generation and MMP disruption in A375 and A2058 cells.

### 3.5. GEF Affects the Expression of Proteins Related to the Apoptotic Pathway

**Figure 4:**
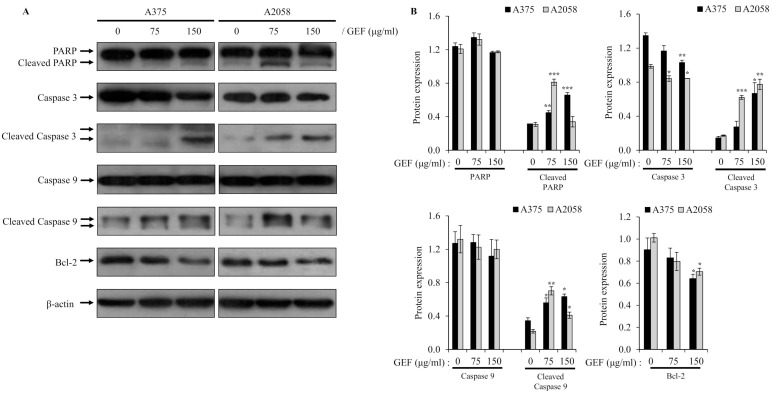
Altered expression of molecules related to apoptosis by GEF in melanoma cells. Cells were incubated with GEF (0, 75, and 150 μg/mL) for 48 h. (**A**) The effect of GEF on protein expression patterns of procaspase-9/-3, Bcl-2, and PARP was examined by Western blotting against β-actin as a loading control. (**B**) Protein expressions were quantified by ImageJ. The data are representative of three independent experiments. The data represent the mean ± SD of three independent experiments. * *p* < 0.05, ** *p* < 0.005, and *** *p* < 0.001.

To understand how GEF induces apoptosis, we examined its effects on the caspase-mediated signaling pathway, which is critical for cell death. Western blot analysis demonstrated activation of caspase-9 and caspase-3 in A375 and A2058 cells treated with GEF for 48 h, along with downregulation or cleavage of Bcl-2 and PARP, which are key targets of activated caspases (Figure 4A,B). These results indicate that GEF triggers apoptosis through caspase activation in melanoma cells.

### 3.6. GEF Inhibits the Growth of A375 and A2058 Xenograft Tumors in Nude Mice

**Figure 5:**
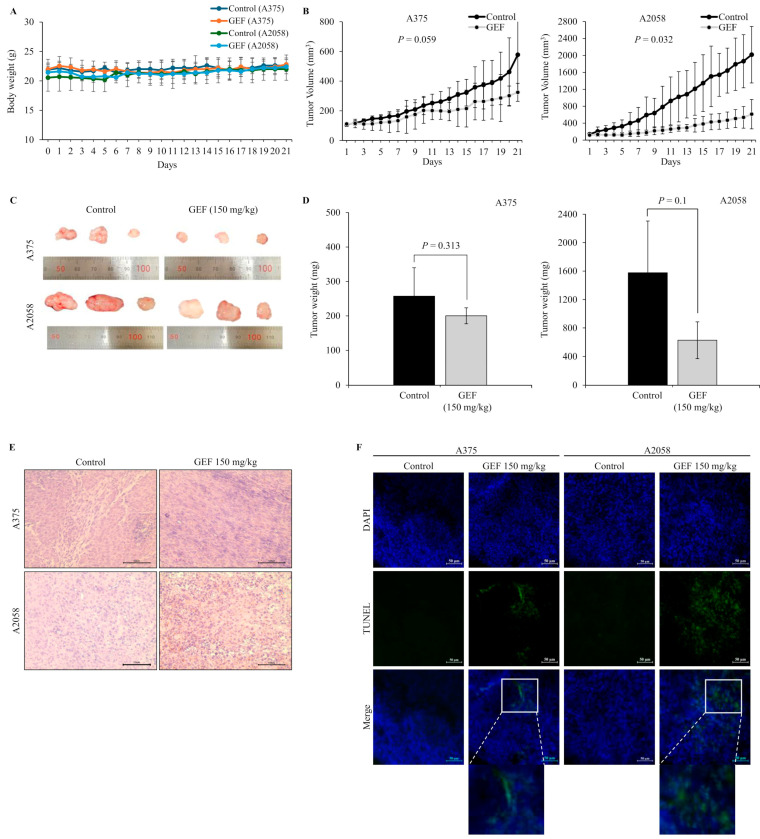
Tumor suppression by GEF in A375- and A2058-xenografted mice. A375- and A2058-xenografted nude mice received oral administration of either control or GEF (150 mg/kg). (**A**,**B**) Body weight and tumor volume were measured over three weeks. (**C**) At week 3, animals were euthanized, and tumors were excised. Representative images of the tumors are shown (for each group, *n* = 3). (**D**) Tumor weights were quantified, and the data are presented as the mean ± SD from three independent experiments, with significant differences denoted by *p* values. (**E**,**F**) Tumor tissue histological analysis was performed using H and E and DAPI/TUNEL staining. Scale bar = 50 μM.

**Figure 6:**
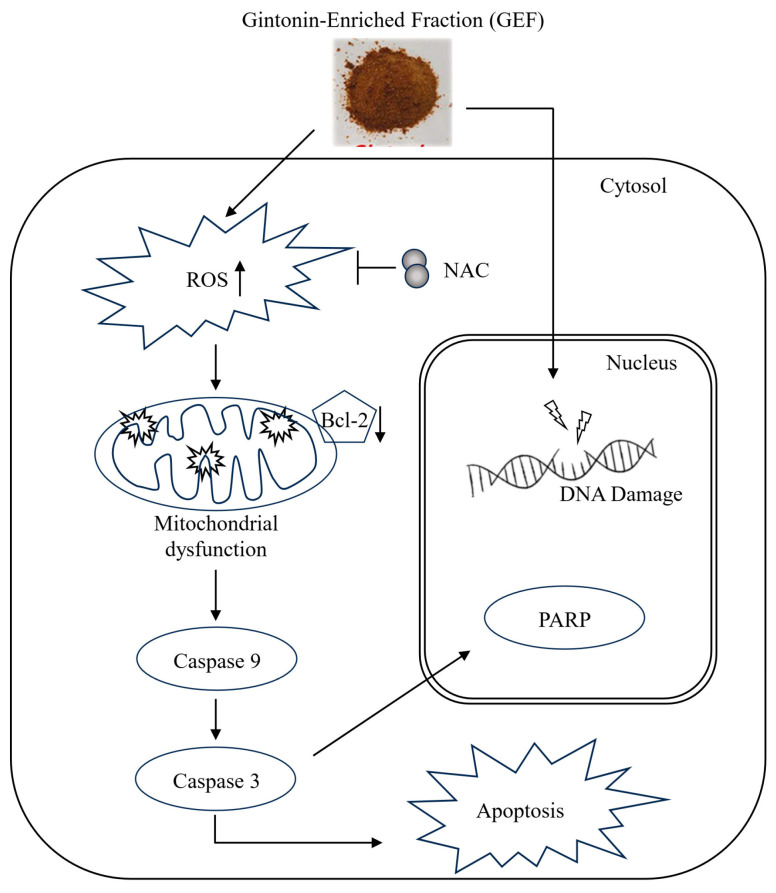
Schematic representation of the signaling pathway responsible for GEF-mediated apoptosis of melanoma cells.

In nude mice implanted with A375 and A2058 cells, GEF treatment did not affect body weight (Figure 5A) but significantly suppressed tumor growth in a time-dependent manner (Figure 5B–D). No signs of toxicity were observed in treated mice. H and E staining revealed smaller atrophic cell shapes in GEF-treated tumor tissues (Figure 5E), whereas TUNEL staining showed increased apoptosis compared with the control group (Figure 5F). These findings support antitumor effects of GEF in vivo, as illustrated in the schematic pathway (Figure 6).

## 4. Discussion

Clinical treatment for advanced melanoma is currently challenging; therefore, it is crucial to develop novel and effective agents for the treatment of patients with melanoma. Ginseng contains high levels of gintonin LPAs. Despite numerous studies on gintonin, its capacity to impede melanoma cell proliferation and the underlying antitumor mechanisms have not been well elucidated.

Recently, we reported that GEF sensitizes renal carcinoma cells to tumor necrosis factor-related apoptosis, inducing ligand (TRAIL)-induced apoptosis [16], showing the combined effects of TRAIL and GEF. A study has suggested that gintonin has potential to suppress melanoma by inhibiting autotaxin activity and limiting tumor growth [40]. However, the precise cellular signaling and molecular mechanisms remain unclear. This study investigated the antitumor properties of GEF in melanoma and revealed its effect on cellular pathways and mechanisms in vitro and in vivo. Our findings highlighted the role of GEF in the induction of apoptosis, cell cycle arrest, and caspase activation in melanoma cell lines.

In vitro experiments revealed that GEF substantially attenuated the proliferation of A375 and A2058 cells. The inhibitory effect ranged from 63.2% to 85.5% in A375 cells and 55.5% to 87.6% in A2058 cells following 24 to 72 h of exposure to 200 μg/mL GEF. Moreover, GEF exhibited negligible cytotoxicity in both cell lines, indicating potent anti-melanoma activity with minimal toxicity and side effects.

Cell cycle progression, which is vital for cell proliferation, involves phases, such as G_0_, G_1_, S, G_2_, and M, which ensure genomic integrity [41]. Upon encountering DNA damage, cells undergo arrest primarily in the G_0_/G_1_ phase to prevent the propagation of defective genetic material into subsequent phases. This arrest allows cells to undergo repair or apoptosis [42,43]. Additionally, the G_2_/M checkpoint ensures fidelity of DNA replication by halting cell division when DNA damage is detected, providing protection against genomic instability [44]. Our findings demonstrated that GEF treatment led to cell cycle arrest, specifically in the G_0_/G_1_ phase in A375 cells and the G_2_/M phase in A2058 cells. Previous studies have shown similar effects with ginsenoside Rg3 that induced G_0_/G_1_ arrest in gallbladder cancer cells [45] and red ginseng-derived panaxytriol that caused G_2_/M arrest [46], highlighting the versatility of gintonin in modulating different cell cycle checkpoints depending on the tumor type. Moreover, our study showed that GEF disrupted colony formation in melanoma cells, indicating its efficacy in hindering melanoma proliferation.

Apoptosis, a programmed cell death mechanism, is activated in cancer cells through both intrinsic and extrinsic pathways, resulting in characteristic cellular changes [47]. Activation involves initiator caspases (caspase-8 and -9) sequentially activating downstream procaspases, leading to executioner caspase activation (caspase-3, -6, and -7) and cell death [47]. Annexin V/PI assay results showed an increased apoptotic cell population in GEF-treated melanoma cells, indicating reduced cell viability. TUNEL staining confirmed DNA fragmentation and nuclear condensation post-GEF treatment. Western blot analysis revealed GEF-induced caspase-9 and -3 activation, PARP cleavage, and Bcl-2 downregulation, suggesting induction of apoptosis by GEF in melanoma cells.

ROS serve as crucial mediators of cell signaling and regulate key apoptotic pathways mediated by mitochondria, death receptors, and the endoplasmic reticulum (ER) [48]. Elevated ROS levels can trigger cell death via the intrinsic apoptotic signaling pathway, characterized by MMP, cytochrome c release, apoptosome formation, and activation of caspase-3 [49,50]. Our findings demonstrate that GEF treatment leads to an increase in intracellular ROS levels. Additionally, ROS-mediated regulation of MMP [51,52] suggests that ROS accumulation and alterations in MMP contribute to GEF-induced cell death.

Oral administration of GEF resulted in the inhibition of A375 and A2058 xenograft tumor growth in nude mice, accompanied by an increase in TUNEL-positive cells in the tumor tissue, indicative of apoptotic cell death compared with the control group. Prior research by Hwang et al. showed similar outcomes consistent with our findings, in which oral administration of gintonin effectively suppressed tumor growth and metastasis in B16/F10 xenograft mice [35]. This study reveals that GEF from ginseng extract exhibits potent antitumor effects by inducing apoptosis and inhibiting the proliferation of melanoma cells in vitro and in vivo.

## Data Availability

The original contributions presented in this study are included in the article/Supplementary Materials. Further inquiries can be directed to the corresponding authors.

## Supplementary Materials

The following supporting information can be downloaded at https://www.mdpi.com/article/10.3390/foods14030381/s1, Figure S1: Effect of GEF on morphological changes and detection of apoptotic cell body in melanoma cell nucleus. Cells were treated with 75 μg/mL and 150 μg/mL GEF or 0.15% DMSO as a control for 24 h. TUNEL and DAPI staining examined under a fluorescence microscope (×200). Experiments were performed in three independent biological replicates and representative images are shown; Table S1: Amount of lipids in GEF.
