# Supplementary material for: Gintonin-Enriched Panax ginseng Extract Induces Apoptosis in Human Melanoma Cells by Causing Cell Cycle Arrest and Activating Caspases
Source: Foods. 2025 Jan 24;14(3):381. doi: 10.3390/foods14030381 (PMC11816885; doi:10.3390/foods14030381)
Supplement: Supplementary file 1 [file foods-14-00381-s001.zip › foods-3402256-supplementary.pdf]

Supplementary Materials

Supplementary Table S1. Amounts of lipids in GEF.

Analyte	Amount (mg/g)	Content (%)
Linoleic acid	71.52 ± 2.38	7.15
Palmitic acid	26.77 ± 0.76	2.68
Oleic acid	15.37 ± 0.29	1.54
LPA C18:2	1.91 ± 5.33	0.19
LPA C16:0	0.60 ± 3.19	0.06
LPA C18:1	0.20 ± 0.39	0.02
LPC C18:2	0.78 ± 4.17	0.078
LPE C18:2 [1)]	0.11 ± 8.13	0.011
LPE C16:0	0.20 ± 2.10	0.02
LPE C18:1	BSL	BSL
LPI C18:2 [1)]	0.88 ± 3.16	0.088
LPI C16:0	0.40 ± 4.38	0.04
LPI C18:1	0.10 ± 5.63	0.010
PA 16:0-18:2	11.72± 0.90	1.17
PA 18:2-18:2	4.60 ± 4.90	0.46
PA 16:0-18:1	1.61 ± 4.46	0.16
PC 18:2-18:2	0.24 ± 1.25	0.024
PC 16:0-18:2	0.27 ± 1.54	0.027
PI 18:2-18:2 [1)]	BSL	BSL
PI 16:0-16:0	BSL	BSL

Amount (mg/g) = mean ± RSD (%) derived from measurements of three distinct white ginseng samples. Abbreviations: BSL, below the sensitivity limit; GEF, gintonin-enriched fraction; LPA, lysophosphatidic acid; LC-MS/MS, liquid chromatography-tandem mass spectrometry; LPC, lysophosphatidylcholine; LPE, lysophosphatidylethanolamine; LPI, lysophosphatidylinositol; MRM, multiple reaction monitoring; PA, phosphatidic acid; PC, phosphatidylcholine; PI, phosphatidylinositol; RSD, relative standard deviation. The quantities of LPE C18:2, LPI C18:2, and PI 18:2-18:2 were measured without the use of standards, and their identification and quantification were performed using MRM transitions. The LC-MS/MS conditions and calibration equations were established based on LPE C18:1, LPI C18:1, and PI 16:0-16:0. [1)] https://pmc.ncbi.nlm.nih.gov/articles/PMC6437394/table/tbl2/?report=objectonly#tbl1fn1.

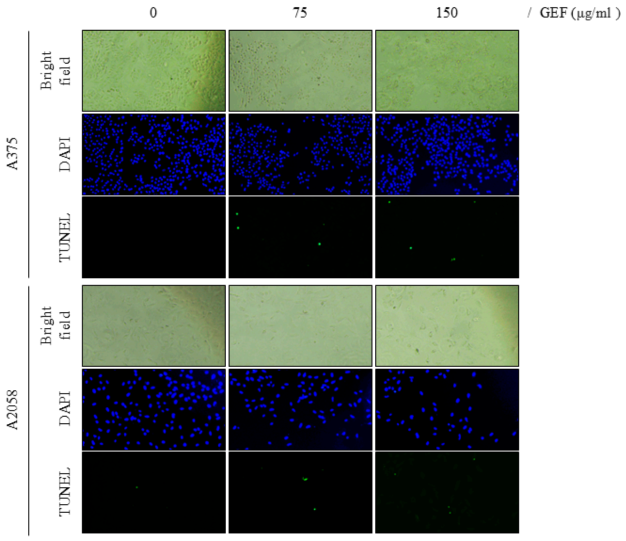

Supplementary Figure S1. **Effect of GEF on morphological changes and detection of apoptotic cell body in melanoma cell nucleus. Cells were treated with 75 μg/ml and 150 μg/ml GEF or 0.15% DMSO as control for 24 h. TUNEL and DAPI staining examined under the fluorescence microscope (×200). Experiments were performed in independent three biological replicas and representative images were shown.**
